# Perceptions of media portrayed violence among South Korean college students using Q methodology

**DOI:** 10.1038/s41598-025-05126-6

**Published:** 2025-06-03

**Authors:** Kang Suk Lee, Song Yi Lee

**Affiliations:** 1 Research Institute for Image & Cultural Contents, Dongguk University, Seoul, Korea; 2 Counseling and Coaching, Dongguk University, Seoul, Korea; 3 Dongguk University, Seoul, 04620 Republic of Korea

**Keywords:** Perceptions, College students, Violence, Media, Well-being, Q methodology, Psychology, Health care

## Abstract

Although numerous studies examine violence portrayed in the media, few have explored how viewers subjectively perceive it despite the presence of contradictory viewpoints. This study employs Q methodology to investigate South Korean college students’ subjective perceptions of media violence. The Q sample comprises 33 statements, and the P-set includes 17 university students. The analysis reveals three perception types. Type 1 (Media Violence Freedom Advocates) believe that personal values influence media violence and do not view it as a problem. They argue that regulating media violence would infringe on freedom of expression. Type 2 (Media Violence Personal Responsibility Emphasizers) view media violence as a means of portraying social issues and find violent content engaging, assuming that wrongdoers ultimately receive punishment. Lastly, Type 3 (Media Violence Ethics and Regulation Advocates) supports regulating media violence and implementing appropriate educational initiatives to address this issue. Understanding these perspectives among college students provides valuable insights for developing media regulations and educational policies.

## Introduction

Media includes movies, over-the-top (OTT) or streaming platforms, television, video games, and YouTube. Technological advancements have increased access to these platforms and provided a broader selection of media content^1^. The media’s influence on individuals’ perceptions of reality is significant, serving as a major conduit for psychological transformation^2^. Although studies on media violence have persisted for decades^3^, recent discussions in South Korea suggest that media exposure may contribute to rising incidents of social violence.

For example, Kim^4^ argues that cyberspace enables users to create alter egos that express exaggerated desires distinct from their real-world selves. These desires can then feed back into reality, raising concerns about the psychological impact of media violence. Beyond promoting aggression, studies show that media exposure can also cause psychological harm^5–7^. Campbell and Valera^8^ found that college students who watched a video on police brutality experienced psychological distress, anxiety, and heightened fear of law enforcement.

Scholars have also explored the social ramifications of media violence, particularly in relation to age and age-based viewing restrictions^9^. Researchers consistently warn that violent content can hinder healthy development in youth^10^. Empirical evidence suggests that violence in media has increased^11^, and technological advancements have made such content more accessible to younger audiences. Consequently, prolonged exposure to violent media may alter prefrontal brain mechanisms involved in regulating behavior, potentially increasing anxiety and stress^12^.

However, not all research highlights negative effects. A meta-analysis by Savage and Yancey^13^ found no correlation between media violence and criminal aggression. Similarly, Drummond et al.^14^ found that aggressive video games have little effect on adolescents’ aggression levels. Some scholars argue that violent media can offer benefits—for example, by exposing the absurdities of societal norms or providing a safe outlet for viewers to process aggression^15,16^. These perspectives suggest that the viewer’s perception and response, rather than the media, may determine the effect.

University students, who regularly engage with violent media across multiple platforms, are particularly important to study. Yet despite the large body of research on media violence, few studies have examined how viewers interpret and internalize that violence, especially given the range of contradictory viewpoints. College students frequently engage with violent media across various platforms, making them a particularly important demographic for study^17^. Among the limited research on this demographic, Lee^18^ surveyed college students’ views on three-dimensional (3D) films to anticipate industry trends. Another study explored college students’ general attitudes toward movie-watching in specific contexts without focusing on genre^19^. However, no study to date has identified or classified how South Korean college students cognitively structure their perceptions of violent media.

In other words, while previous research emphasized quantitative generalization of media violence^14,20,21^, they have largely overlooked the psychological dimensions of how young people perceive it. No research has thoroughly examined how college students—the primary consumers of such media—think about or react to media violence. Devilly et al.^22^ posit that personality traits and frustration levels constitute more robust predictors of anger and aggression than media exposure. Therefore, exploring college students’ subjective perceptions of media violence can yield valuable insights into how they process violent content. This understanding can inform the development of more targeted media regulations and educational policies.

Thus, this study addresses the following research questions:

1. What are the perceptions of South Korean college students regarding the media’s depiction of violence?
2. What are the distinguishing features of South Korean college students’ perceptions of violence depicted in the media?

## Study method

Q methodology uses an inductive approach to analyze significant intra-individual differences and examine how people form subjective perceptions^23^. This method enables researchers to understand how individuals categorize and express their views on a specific issue, allowing for the identification of distinct viewpoints. In doing so, Q methodology uncovers the primary perspectives within a group^24^ and provides a unique approach to highlight and categorize marginalized individuals or ideas, whether intentionally or unintentionally excluded^25^.

**Fig. 1:**
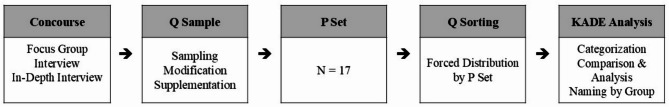
Study process

This study employs Q methodology to reveal the previously marginalized and undisclosed subjective perspectives of college students regarding media violence. Figure 1 illustrates the study process, which begins with developing a concourse—a collection of statements reflecting college students’ shared thoughts and feelings about violence in the media. From this concourse, we selected a representative and comprehensive Q sample relevant to the topic and target group. We then asked participants (the P set) to complete a Q sort, where they categorized the statements based on their viewpoints.

In Q methodology, the emphasis lies on the number of items in the Q sample, not the number of participants. Like qualitative research, Q methodology is for small participant groups^26^.

### Concourse

The concourse is crucial in Q methodology as it captures the range of perspectives on a specific topic. Researchers typically create the concourse through interviews^27^. Thus, to define the population of subjective statements for this study, we conducted a focus group interview with six college students (three females and three males) and in-depth interviews with two additional students (one female and one male), all enrolled at D University. The focus group included four first-year students, one third-year student, and one fourth-year student. The in-depth interviews involved one fourth-year and one third-year student.

We recruited participants by posting an announcement on D University’s eClass online platform, targeting students enrolled in general education courses. From those who expressed interest, we selected participants for focus groups and in-depth in-person interviews. Each in-depth interview lasted about one hour, and we compensated participants with approximately USD 15.

To ascertain students’ overall perspectives on media portrayals of violence, we employed semi-structured interview questions such as, “What are your thoughts on violence portrayed in the media,” “What are your thoughts on the perpetrators of violence portrayed in the media,” “What are your thoughts on the victims of violence portrayed in the media,” “How would you define the violence portrayed in the media,” and “If you believe the violence portrayed in the media should change, why?”

This process generated 121 statements for the concourse. We confirmed data saturation through repeated responses across interviews.

### Q sample

The Q sample consists of a set of statements that represent a subset of the concourse, i.e., the full range of subjective viewpoints on a given topic. Researchers typically include 30 to 60 statements in a Q sample. Unlike traditional survey items designed to measure specific variables or constructs, Q sample statements aim to capture a wide range of perspectives related to the research topic^28^.

To create the Q sample for this study on perceptions of violence portrayed in the media, we began by reviewing the 121 statements gathered in the concourse. From this pool, we initially selected 80 statements. Through collaborative discussion, we refined the list and ultimately selected 43 statements that best captured a wide range of subjective viewpoints. This process yielded 33 statements reflecting a comprehensive and balanced representation of the topic.

### P set

Researchers select the study participants, i.e., the P set, to perform Q sorting based on their relevance to the research topic. Although Q methodology can involve between 10 and 100 participants^29^, its purpose is not to generalize findings but to explore typologies of perception. Because it focuses on attitudes and opinions, it often includes more statements than participants. Despite its small sample size, Q methodology can produce robust and meaningful results^30–32^.

To ensure diversity within the P set, we used purposive sampling to select 80 students enrolled in liberal arts courses at D University. From this group, 17 individuals (6 females and 11 males) participated in the Q-sorting process. We aimed for a balanced mix of majors, academic years, and genders by selecting students from general education courses.

After obtaining consent, we provided participants with instructions to complete the Q sort at home and return it to the researchers. Each participant received approximately USD 10 as compensation.

The final group of six first-year students, five third-year students, and six fourth-year students, all studying at D University in Seoul. We conducted the study between November 20 and 30, 2023.

### Q sorting and data analysis

**Fig. 2:**
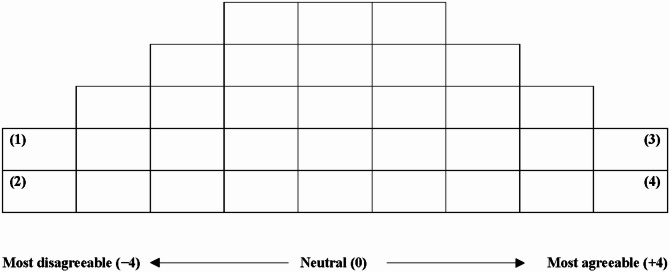
Q-sorting grid.

Q sorting measures the degree to which study participants (the P set) agree or disagree with each statement by having them consider all items together along a continuum. This process enables researchers to identify and compare participants’ perspectives on media violence. Participants arrange the statements on a Q grid based on their level of agreement or disagreement (see Fig. 2).

We analyzed the Q-sorting data using KADE. 1.2.1, a desktop application designed for Q methodology analysis. The program is fully compatible with Microsoft Windows, Apple macOS, and Linux operating systems^33^. For data analysis, we applied principal component factor extraction followed by varimax orthogonal factor rotation. We interpreted the results using factor arrays, which provide z-scores for each statement.

For deeper analysis, we examined the factor scores and Q-sort values for each perspective type. We also reviewed participant’s written explanations for the two statements with which they most strongly agreed and disagreed, enhancing the depth and clarity of our interpretation^23^.

## Results

### Analysis of results

Three distinct factors emerged from the analysis.

Table 1 shows the factor score correlations. The low correlations indicate that the three perspectives are relatively independent. However, because Q methodology emphasizes the discovery of viewpoints rather than assuming complete independence between them, variations in correlation coefficients did not affect the factor extraction process^23^.

**Table 1:** Factor score correlations.

	Factor 1	Factor 2	Factor 3
Factor 1	1	0.3368	0.3506
Factor 2	0.3368	1	0.1699
Factor 3	0.3506	0.1699	1

Table 2 presents the factor scores alongside the corresponding Q-sort values for each factor. A Q-sort value reflects how a participant ranked each statement within a structured distribution, typically shaped like a normal curve.

**Table 2:** Factor scores and Q-sort values of each factor.

No.	Statements	Factor 1		Factor 2		Factor 3	
		Z-score	Q-sort value	Z-score	Q-sort value	Z-score	Q-sort value
S1	I believe media violence is a problem because, regardless of media ratings, the level of violence is severely high.	−1.202	−3	−0.483	−1	0.545	1 *
S2	I feel that media violence is exaggerated compared to reality and that it feels much more cruel.	−0.184	0	−0.729	−1	0.492	1
S3	Without violence, the content of the media feels boring.	−1.229	−3	−1.209	−3	−0.928	−2
S4	The fun factor is much stronger when the characters are positioned equally in a violent scene.	0.885	2 *	−0.482	−1	−0.491	−1
S5	Violence feels overly brutal when there is a power differential between the perpetrator and the victim.	0.890	2	0.252	0	0.605	1
S6	If it is not real, I would like to play the role of a perpetrator of violence.	−1.832	−4	−1.664	−3	−1.616	−4
S7	I feel that violence is good when you see the perpetrator get violently harmed.	−0.913	−2	1.056	2 *	−0.795	−1
S8	I do not want to watch the movie if there are violent scenes.	−1.905	−4	−1.213	−3	0.278	0 *
S9	Unfortunately, so many movies with violent scenes are successful.	−0.923	−2	−1.010	−2	0.168	0 *
S10	Violence in zombie movies and dramas is not scary.	0.274	0	0.873	2	−0.271	−1
S11	I think violence is necessary if it adds to the value of the media content.	−0.472	−1	0.898	2 *	−0.817	−2
S12	I do not think media violence has an impact on society.	−0.540	−1*	0.289	0*	−1.403	−3*
S13	I feel that if people think violence in media is a problem, they should not watch it.	1.495	4	1.369	3	−0.384	−1*
S14	I think children should not watch murder scenes.	1.389	3	0.674	1	1.923	4
S15	The presence, absence, or level of violence is not a criterion for media selection.	0.554	1	1.541	4*	−0.193	0
S16	Watching violent scenes can also be a new experience.	1.161	3*	−0.057	0 *	−0.922	−2*
S17	I think that the more we watch media violence, the more we become desensitized to real-life violence.	1.098	2	−1.781	−4*	0.590	1
S18	If the content of the violent scene somehow connects to me, I feel that I take it more seriously.	0.892	2	0.380	1	0.986	3*
S19	I think producers should not portray cruel scenes like murder in detail.	−0.003	0	−0.167	−1	1.796	4*
S20	I think that media violence is getting worse because of OTT.	0.771	1	−1.793	−4*	0.890	2
S21	Sometimes, I think that violence is exaggerated to the point where it breaks the flow of the story.	0.136	0	0.797	2	1.179	3
S22	The content with violence is interesting because their premise is to punish bad people.	0.376	1	1.482	4*	0.396	1
S23	I think that violence in media plays an essential role in showing social issues.	−0.596	−1	1.282	3*	−0.223	0
S24	I believe that media violence serves as a cathartic release mechanism.	0.828	1*	−0.928	−2	−1.287	−3
S25	Movies and TV dramas with violence based on true stories are entertaining.	−0.698	−1	0.304	1*	−1.313	−3
S26	I believe we should provide an appropriate education program regarding media violence.	0.677	1	0.201	0	1.655	3*
S27	When I watch media violence, I tend to empathize more with the victims.	−0.194	0*	0.606	1	0.664	2
S28	If the level of violence decreased, I would watch more media.	−1.030	−3	−1.032	−2	−0.174	0
S29	I want to watch violent media when I am stressed.	−0.882	−2	−0.876	−2	−1.675	−4*
S30	I think the media should not cover suicide.	−0.894	−2	−0.688	−1	−1.069	−2
S31	I think the media should also cover the consequences of violence.	1.269	3	1.293	3	0.814	2
S32	I believe that regulating violence in media violates freedom of expression.	1.629	4*	0.271	0	−0.378	−1
S33	When I watch a violent scene that seems unnecessary, I feel uncomfortable.	−0.826	−1*	0.546	1	0.959	2

*p < 0.05.

Table 3 shows the factor matrix, including flagged defining sorts. These flagged entries identify participants whose Q sorts strongly align with a specific factor type.

**Table 3:** Factor matrix with flagged defining sorts.

*P*-Sample	Sex	Grade	Major	Factor 1		Factor 2		Factor 3	
1	F	1	Artificial Intelligence	0.2934		−0.1727		0.6825	Flagged
2	M	3	Multimedia	−0.0480		0.5857	Flagged	−0.0727	
3	M	4	Drama	0.2534		0.7572	Flagged	−0.2464	
4	M	1	Law	0.3623	Flagged	0.0740		0.2960	
5	F	3	Japanese Studies	0.7651	Flagged	0.0281		0.1664	
6	M	4	Economics	0.1888		0.6967	Flagged	0.0566	
7	M	1	Business Administration	−0.0242		0.8443	Flagged	0.3040	
8	M	1	Advertising and Public Relations	0.6944	Flagged	0.2754		−0.0233	
9	M	1	Police Administration	0.3351		0.4818	Flagged	0.0270	
10	F	1	Police Administration	0.2321		0.0850		0.6973	Flagged
11	F	3	Business Administration	−0.1403		−0.2059		0.7832	Flagged
12	M	4	International Trade	0.0698		0.1788		0.7204	Flagged
13	M	4	Electronic and Electrical Engineering	0.6227	Flagged	0.3526		−0.3455	
14	M	4	International Trade	0.7914	Flagged	0.0837		0.3097	
15	M	3	Law	0.7914	Flagged	0.0837		0.3097	
16	F	3	English Language and Literature	0.2557		0.2404		0.8335	Flagged
17	F	4	Motion Picture & Visual Arts	0.1671		0.6941	Flagged	0.1434	
% Explained Variance				19		19		20	

Note. Flagged: The analysis of research participants’ Q-sort results in relation to key factors and their association with specific factors.

#### Factor scores for type 1: media violence freedom advocates

Type 1 participants view media violence as a matter of free expression. They strongly agreed with statements such as S32: “I believe that regulating violence in media is a violation of freedom of expression”(Q-sort value = + 4), S13: “I feel that if people think violence in media is a problem, they should not watch it,” S16: “Watching violent scenes can also be a new experience,” and S31: “I think the media should also cover the consequences of violence.” In contrast, they strongly disagreed with S8: “If there are violent scenes, I do not want to watch the movie,” and S6: “If it is not real, I would like to play the role of a perpetrator of violence.”

P15, a participant in this group, emphasized the value of personal judgment and firsthand experience: “I think that preventing approaching violence too extremely can be counterproductive and that the experience of violence can also form correct values.” Similarly, P5 said, “I think we should prevent young children from watching violent scenes, but I do not want to watch movies with violent scenes as part of the overall content.”

These comments reflect a nuanced stance: while participants support limiting exposure for children, they believe adults should have the freedom to choose what they consume. They argue that media violence, when contextually justified, is a legitimate form of expression that does not automatically result in real-world aggression. Instead, they see it as an opportunity for exploration and reflection.

Based on these views, we labeled this group as “Media Violence Freedom Advocates.”

#### Factor scores for type 2.: media violence personal responsibility emphasizers

Type 2 participants view exposure to media violence as a matter of individual choice and personal responsibility. They strongly agreed with statements such as S15: “The presence, absence, or level of violence is not a criterion for media selection”(Q-sort value = + 4), S22: “The content with violence is rather interesting because their premise is to punish bad people,” S7: “I feel that violence is good when you see the perpetrator get violently harmed,” and S23: “I think that violence in media plays an important role in showing social issues.” In contrast, they strongly disagreed with S20: “I think that media violence is getting worse because of OTT,” and S17: “I think that the more we watch media violence, the more we become desensitized to real-life violence.”

P3 explained, “The criteria for media selection are affected by personal tastes and interests, and if the content needed in the media is violent, it is not uncomfortable to watch it.” P17 added, “Viewers who watch media can choose what they like, and media providers can handle violence for cinematic quality according to their intentions. Therefore, viewers need the wisdom to make wise choices about violent content.” P17 emphasized that the portrayal of violence in the media is meaningful in addressing social issues or moral lessons, such as punishing wrongdoing.

This group believes that the responsibility for consuming violent media lies with the viewer, not the media. They argue that violent content does not inherently cause harm and can be enjoyable and meaningful when it aligns with narrative or artistic intent. Rather than condemning media violence, they focus on the viewer’s ability to critically assess and choose what to watch.

For these reasons, we classified this group as “Media Violence Personal Responsibility Emphasizers.”

#### Factor scores for type 3: media violence ethics and regulation advocates

Type 3 participants view media violence as a social harm. They strongly agreed with statements such as S14: “I think children should not watch scenes that involve murder”(Q-sort value = + 4), S19: “I think producers should not portray cruel scenes like murder in detail,” S1: “I believe media violence is a problem because regardless of media ratings, the level of violence is severely high,” and S26: “I believe we should provide an appropriate education program regarding media violence.” In contrast, they strongly disagreed with S29: “I want to watch violent media when I am stressed,” and S12: “I do not think media violence has an impact on society.”

P16 stressed the emotional harm caused by violent media, stating, “If encountered in childhood, violent scenes such as murder scenes should have a very negative effect emotionally. Even in the case of adults, it is important to educate them about the dangerousness of media violence.” Similarly, P11 expressed concern about indiscriminate exposure to violent content, saying,

I think there are many cases where people view violent scenes indiscriminately in short videos, such as shorts recently, and those who supply media videos should be careful about this. Moreover, the violence covered in the media concurs with the violence that occurs in society.

This group believes that viewers and content creators have a responsibility to handle violent media with caution. They argue that unrestricted exposure to graphic content can lead to desensitization and emotional harm, particularly among younger audiences. As such, they advocate for stronger ethical standards, educational efforts, and regulatory measures.

For these reasons, we labeled this group as “Media Violence Ethics and Regulation Advocates.” They hold a highly negative view of media violence, actively avoid such content, and emphasize its potential to cause real-world harm.

## Discussion

This study examined and categorized college students’ perceptions of violence portrayed in the media and identified three types.

Although the three types emphasize different aspects, several statements reveal a common concern across all groups about the negative effects of irresponsibly presented media violence. This suggests that, despite their differences, participants share a fundamental ethical stance on media violence.

Type 1, the “Media Violence Freedom Advocates” group, believes that media violence has a positive function and values its expression as a form of free speech. While various studies have addressed the problematic nature of violent media content^34,35^, this group prioritizes freedom of expression and individual autonomy over such concerns. However, Moon^36^ emphasizes the need to balance freedom of expression with civic responsibility. Type 1 participants would benefit from opportunities to discuss the meaning of balance from their perspective, particularly in terms of individual choices and efforts required to maintain such equilibrium. Educators should also inform them about research linking exposure to violence to increased violent behavior.

Type 2, the “Media Violence Personal Responsibility Emphasizers” group, supports the inclusion of violence in media when it serves the narrative or artistic purpose, particularly from the creators’ perspective. As viewers, they emphasize the importance of making informed, interest-based choices. They value the role of media in addressing social causes and see violence as justified when it conveys moral lessons, especially when it involves punishing wrongdoing. This observation aligns with Lee and Suh’s^37^ findings regarding school violence, which found that Korean college students often show emotional empathy toward punishing perpetrators and possess a strong moral awareness regarding victim protection and violence prevention. Type 2’s views reflect cultural characteristics specific to South Korea.

While Type 1 and Type 2 hold neutral or even positive attitudes toward media violence, they differ in important ways: Type 1 opposes regulation altogether, whereas Type 2 supports violent content only when it serves a clear narrative or social function.

Type 3, the “Media Violence Ethics and Regulation Advocates” group, holds the most critical view of media violence. They call for stricter regulation by media providers and believe viewers must recognize the potential psychological and societal harm of violent content. They also advocate for educational programs that raise awareness of these risks. Si^38^ shares a similar position, stating that viewers must learn to identify, reject, and avoid violent content through structured education. Similarly, Busching and colleagues^39^ highlight the negative psychological effects of media violence, such as increased aggression and attention deficits. They argue that no evidence supports positive outcomes. Type 3’s concerns about media violence translating into real-world behavior align with Juhi’s^40^ findings, which show a connection between watching violent movies and aggressive behaviors.

This study identified three types of perceptions regarding media violence. Busching et al.^39^ found that the effects of media violence depend on content characteristics, the individual viewer, and broader social influences. Kubrak^41^ reinforces this by demonstrating that graduate and undergraduate students respond differently to the same media, suggesting that individual interpretation plays a greater role than the content. Kubrak’s findings support the typologies revealed in the present study.

Moreover, Prot and colleagues^42^ argue that media violence affects individuals regardless of cultural background. However, a comparative study between South Korean and Chinese college students^37^ found differing interpretations of violent themes, suggesting that while media violence may have universal effects, culture significantly shapes its meaning.

Notably, students from the drama and visual art departments in our study tended to align with Type 2, indicating that those involved in media creation are more likely to consider the intent behind violent content. This finding supports the notion that perceptions of media violence vary according to individuals’ interests, disciplines, and professional orientations.

### Study implications

This study offers several key implications.

First, it highlights the diverse ways college students perceive media violence, emphasizing the need for tailored educational approaches that address these varied viewpoints.

Second, it emphasizes the importance of striking a balance between media regulation and freedom of expression. This study offers policy recommendations and calls for open discussions on the appropriate scope of regulation.

Third, by examining the effects of media violence on personal behavior and societal attitudes, the study advocates for media education that fosters critical thinking and social responsibility.

Fourth, the study examines how students’ perceptions influence their media consumption habits. It recommends educational strategies that foster healthier, more intentional engagement with violent content.

Ultimately, the study proposes a comprehensive approach to media violence, one that combines ethical regulation with educational interventions to address the issue holistically.

### Limitations

While this study explored college students’ perceptions of violence portrayed in the media, drawing participants from various grades and majors, second-year students showed less interest in participating. This lack of engagement led to an incomplete representation across grade levels.

Additionally, the study did not clearly distinguish between mass media and social media platforms. Future research should investigate the psychological effects of media violence within each type of media to provide more refined and targeted insights.

## Conclusion

This study identified three distinct perspectives on media violence among college students, highlighting the need for more nuanced and targeted policy interventions. Type 1 (“Media Violence Freedom Advocates”) values cultural norms that prioritize freedom of expression. Type 2 (“Media Violence Personal Responsibility Emphasizers”) emphasizes individual autonomy in media consumption and supports narratives where justice is served. Type 3 (“Media Violence Ethics and Regulation Advocates”) considers media violence a critical societal issue and strongly supports both regulatory frameworks and educational initiatives.

Despite their differing orientations, all three groups demonstrated an implicit consensus that irresponsibly portrayed media violence particularly when devoid of ethical framing or consequences can be problematic. This shared concern underscores the importance of embedding ethical considerations into violent media, regardless of viewers’ interpretive frameworks.

Rather than classifying media violence as simply harmful or harmless, these findings reveal the nuanced and multifaceted nature of students’ subjective perceptions. Acknowledging these diverse viewpoints enriches the field of media and communication studies and offers actionable insights for the design of educational programs and policy strategies that better resonate with the media experiences of today’s youth.

Furthermore, this study underscores the methodological value of Q methodology as a powerful complement to traditional survey techniques. While surveys are useful for identifying general patterns, they often miss the layered and sometimes contradictory nature of individual attitudes. In contrast, Q methodology captures distinct configurations of thought, making it particularly effective for exploring complex psychosocial phenomena such as perceptions of media violence.

## Data Availability

The datasets generated and/or analysed during the current study are not publicly available to preserve the anonymity of the respondents but are available from the corresponding author on reasonable request.
